# Green synthesis of novel spiropyrazoline-indolinones in neutral deep eutectic solvents and DFT studies

**DOI:** 10.1016/j.heliyon.2023.e23814

**Published:** 2023-12-20

**Authors:** Zubi Sadiq, Ambreen Ghani, Muhammad A. Hashmi, A. Dahshan, Shahnaz, Samiah H. Al-Mijalli, Munawar Iqbal, Erum A. Hussain

**Affiliations:** a Department of Chemistry, Lahore College for Women University, Lahore, 54000, Pakistan; b Department of Chemistry, University of Education Lahore, Vehari Campus, 61100, Pakistan; c Department of Chemistry, University of Education, Attock Campus, Attock, 43600, Pakistan; d Department of Physics, Faculty of Science, King Khalid University, P.O. Box 9004, Abha, Saudi Arabia; e Department of Biology, College of Sciences, Princess Nourah bint Abdulrahman University, P.O. Box 84428, Riyadh, 11671, Saudi Arabia; f Department of Chemistry, Division of Science and Technology, University of Education, Lahore, Pakistan

**Keywords:** Spiropyrazoline, Deep eutectic solvent, Intramolecular cyclization, Density functional theory

## Abstract

Novel spiropyrazoline-indolinones (**4a–t**) have been synthesized successfully in neutral deep eutectic solvents by reacting 5-Cl/Br-isatin (**1a–b**) with aromatic ketones (**2a–b**) and a variety of substituted hydrazines (**3a–e**) in good to excellent yields. This eco-friendly straightforward synthetic protocol discloses good functional group compatibility. The conventional synthetic approach was compared with the greener route of microwave-assisted synthesis of spiropyrazolines using ethanol. This approach utilized mild reaction conditions which furnished high yields in short reaction time employing one pot two-step multicomponent. All new compounds were structurally confirmed by detailed spectroscopic analysis and density functional theory calculations. This method provides efficient access to spiropyrazole derivatives using biodegradable and green solvent.

## 1 Introduction

Spirocyclic compounds are an attractive target in drug discovery due to their enriched bio-profile [[1], [2], [3]]. These molecules are widely distributed in natural and non-natural products and show impressive biological properties [[4], [5], [6]]. Other applications of spirocyclic molecules include asymmetric synthesis and organic optoelectronics [1,7]. In the case of spiropyrazoline, two rings are connected at the C-5 position [8,9] with a unique spirocyclic junction that is conformationally rigid [1,10]. Due to widespread applications of these spiro skeletons, effective methods have been developed including catalyst-assisted synthesis [11], catalyst-free synthesis [12], cycloaddition reaction using diaziridines and nitrile imines or diazoalkanes, as appropriate dipoles where multistep reaction sequence is adopted and at times suffers poor yield and regioselectivity [7,13]. Even with these inspiring advances, it is still important to further investigate better synthetic approaches for spiropyrazolines under the umbrella of green chemistry.

**Fig. 1:**
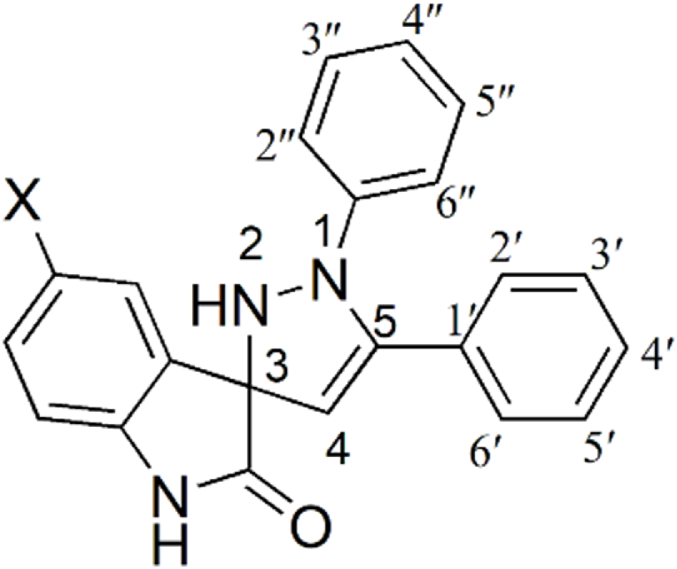
A representative structure of spiropyrazoline-indolinones.

Because of environment concerns, non-toxic, biodegradable and recyclable solvents like deep eutectic solvents (DES) are emergent solvents for synthesis [[14], [15], [16], [17]]. Additionally, these solvents are less volatile, biocompatible, cost-effective, water insensitive and easy to prepare with no purification issues and storage [[18], [19], [20], [21]]. Inspired by these features, the present research aims to endeavor the synthesis of new spiropyrazolines in DES as no attempt has been made before to the best of our knowledge. Moreover, a comparison is also made between the convection heating and microwave heating methods. Synthetic chemists execute post-reaction amendments that allow molecular complexity in core scaffolds for various improved applications. This leads to the region and stereo-specific frameworks with functional diversity. Thus, to accomplish this effectively, a multicomponent reactions (MCR) approach is employed for the synthesis of spiropyrazolines. Therefore, tethering the 1,2-pyrazoline and indolinones motifs simultaneously into one molecule as spiropyrazoline-indolinones (Fig. 1) seems to be a potential strategy for drug discovery.

Organic synthesis is useful for drug design when it is combined with computational chemistry to study various electronic parameters of compounds, hence, provides very valuable information [22]. Apart from the geometries of compounds, the picture of molecular size and its electronic structure can be studied with the help of density functional theory (DFT) calculations. They provide detailed information about the frontier molecular orbitals (FMOs) of all the compounds. This study was performed using the PBE0/def2-TZVP basis set to achieve an understanding of their structural properties. Similarly, using Frontier Molecular Orbital (FMO) analysis, the kinetic stability of all compounds (4a-4t) was calculated at the same level of theory. Molecular electrostatic potential (MEP) mapping over the entire stabilized geometries of the molecules was used to determine reactive sites. Furthermore, the PBE0/def2-TZVP level of theory was used to simulate the first hyperpolarizability analysis, which provided insights into nonlinear optical response.

## 2 Materials and methods

All chemicals were purchased from Merck, Sigma Aldrich, Riedel-de Haën and used without purification. Reactions were monitored through pre-coated TLC plates of silica gel (Merck 60 F254, 0.2 mm thick) and spots were examined under UV light (CAMAG Scientific Inc) at 254/365 nm. Column chromatography was used to purify compounds over Merck silica gel 60 (0.063–0.200 mm, 70–230 mesh). MW-assisted synthesis was conducted in CEM Discover microwave reactor, model 908010, volts 180/264 VAC, max. power 300W (Synergy software). Melting points were recorded on digital Stuart apparatus, SMP 10 model and considered uncorrected. FTIR spectra were mentioned in wave numbers (cm^−1^) using Alpha ATR-IR spectrometer, Bruker. ^1^HNMR spectra were determined at AVII400, AV400RG (400 MHz) and ^13^CNMR spectra were recorded at 100.56 MHz on a Bruker spectrophotometer. Chemical shift (*δ*) values are noted in ppm and coupling constants are indicated as *J* values in Hz. Characterization of the signal fragmentations: s = singlet, d = doublet, dd = doublet of doublet, t = triplet, q = quartet, m = multiplet. Elemental analyses were performed on the Perkin Elmer 2400 CHNS elemental analyzer.

### 2.1 Preparation of deep eutectic solvents (DES)

Three different DES was prepared according to the literature method with little modifications [[23], [24], [25]].

#### 2.1.1 DES-1

Choline chloride (100 mmol, 13.96 g, 1 eq.) along with urea (200 mmol, 12 g, 2 eq.) were slowly heated at 80 °C for 10 h to obtain a colorless homogenized solution. This solution was cooled at room temperature and used for synthesis without any further purification. pH: 7, Freezing point: 12 °C, FTIR cm^−1^: 783 (N–H), 864 (C–N), 1475 (CH_2_), 1609 (N–H), 1662 (C=O), 3189 (O–H and N–H), 3321 (NH_2_).

#### 2.1.2 DES-2

Choline chloride (100 mmol, 13.96 g, 1 eq.) along with fructose (100 mmol, 18 g, 1 eq.) was gently heated at 50 °C to get a light-yellow adhesive solution after 7 h. This solution was cooled at room temperature for synthetic purposes without purification. pH: 7, Freezing point: 5 °C, FTIR cm^−1^: 570 and 3200 (OH), 862 and 1042 (C–C–O), 1120 (C–O), 1205 (C–O–H), 1470 (CH_2_), 1655 (C=O), 2870 and 2900 (C–H), 3012 (N–H).

#### 2.1.3 DES-3

Choline chloride (100 mmol, 13.96 g, 1 eq.) was slowly heated with glycerol (200 mmol, 18.4 g, 14.6 mL, 2 eq.) at 110 °C for 8 h. The resultant colorless solution was cooled at room temperature for synthetic purposes without further purifying. pH: 7, Freezing point: −40 °C, FTIR cm^−1^: 565 and 3300 (OH), 862 and 1036 (C–C–O), 1110 (C–O), 1205 (C–O–H), 1485 (CH_2_), 2876 and 2932 (C–H), 3035 (N–H).

### 2.2 Procedure

#### 2.2.1 DES mediated synthesis of spiropyrazoline-indolinones

To a stirred mixture of 5-chloro/bromo isatin (**1a–1b**, 5 mmol, 1 eq.), aromatic acetyl ketones (**2a–2b**, 5 mmol, 1 eq.) and diethyl amine (6.5 mmol, 671 μL, 474 mg, 1.3 eq.) at room temperature, then after TLC monitoring, hydrazine derivative (**3a–3e**, 5 mmol, 1 eq.) was added with DES-1 (8 mL) and heated at 65 °C till the completion of reaction. The flask's contents were poured over crushed ice to induce precipitation, which was then neutralized with 10% glacial acetic acid. To obtain purely targeted spiropyrazolines, the crude product was subjected to column chromatography using an eluting system with ethyl acetate and n-hexane as a gradient.

#### 2.2.2 Microwave-assisted synthesis of spiropyrazoline-indolinones

An equimolar mixture of 5-chloro/bromo isatin (**1a/1b**, 3 mmol), acetyl ketones (**2a**/**2b**, 3 mmol) and different hydrazine derivatives (**3a–3e**, 3 mmol) were introduced in a Teflon reaction vessel equipped with diethyl amine (3.9 mmol, 403 μL, 285 mg, 1.3 eq.) in solvent ethanol (5 mL). This mixture was irradiated for 8–17 min at 240 W. Infrared (IR) temperature was maintained at 70 °C having the pressure of 20 bar inside the reaction vessel throughout the synthesis. Reaction progress was monitored by TLC at an interval of 30 s using ethyl acetate: *n*-hexane (4:6 v/v) as eluent. The work-up procedure was similar as mentioned in the conventional setup previously.

## 3 Results and discussion

### 3.1 Chemistry of spiropyrazoline-indolinones

**Scheme 1:**
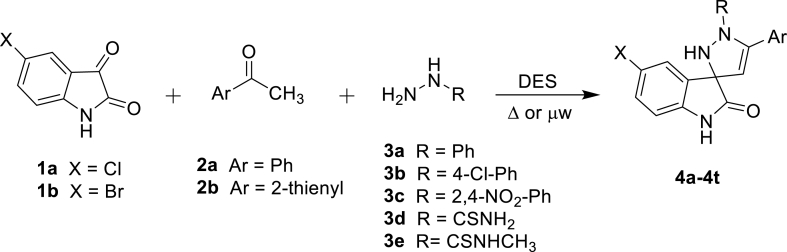
Construction of spiropyrazoline-indolinones from halo-substituted isatin.

New spiropyrazoline-indolinones **(4a–4t)** were synthesized using DES as a catalyst in addition to its role as reaction media. The multicomponent reaction proceeded with 5-Cl or 5-Br isatin (**1a/1b**), acetophenone or 2-acetyl thiophene (**2a**/**2b**) and different hydrazine analogs (**3a–3e**) in the presence of diethyl amine (DEA). In general, the multicomponent reaction proceeded leading to the cyclization step. According to available protocols [26,27], initially two steps of synthetic protocols were followed which were low yielded due to purification issues. These unsatisfactory outcomes were our motivation to explore one-pot, two-steps strategy for spiropyrazolines synthesis which sounds promising. In the first step, exocyclic *α,β*-unsaturated ketone (**I**) was produced by the reaction of **1a/1b** with acetyl ketone **2a/2b**. In the second step, enone (**I**) and selected hydrazine derivatives (**3a–3e**) were reacted to furnish a desired product which underwent column purification as mentioned in Scheme 1.

**Fig. 2:**
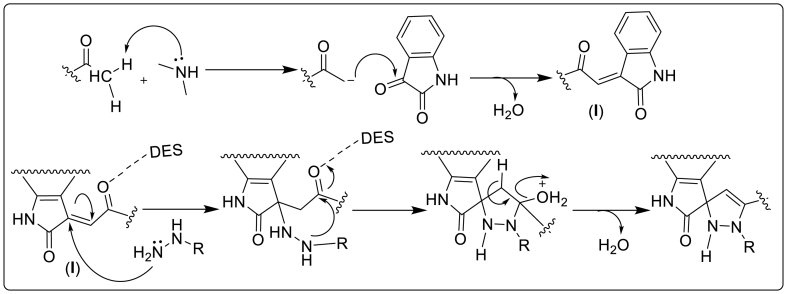
A plausible reaction mechanism in DES.

The plausible reaction mechanism showed Aldol condensation in the first place then Michael addition reaction to yield the final product [9,22,28]. In the Aldol reaction, DEA acts as a base to abstract acidic proton from acetophenone to yield a reactive anionic intermediate which quickly reacts with the carbonyl group of isatin to furnish a stable *α,β*-unsaturated ketone (**I**). The exocyclic C=C of enone is attacked by nitrogen of hydrazine analogous to Micheal addition to start a reaction series that furnishes pyrazoline ring finally. DES played an important role in catalyzing via hydrogen bonding with carbonyl oxygen (**I**), thus making it more electrophilic and providing the ease of nitrogen attack to form a spiro junction (Fig. 2). The reaction conditions favored the aldol mechanism as it occurs at room temperature while another possibility is to achieve formation via Schiff ‘s base route, however, it requires elevated temperature.

#### 3.1.1 Optimization of reaction conditions

Initially, the reaction between isatin, acetophenone and thiosemicarbazide was chosen for optimization studies. For this, the equimolar ratio of reactants was treated in the presence of 1 mmol of different suitable bases in ethanol and diethyl amine (DEA) formed the most appreciable amount of product (Table 1, entry 4). Initially isatin reacted with acetophenone to give a yellow mixture that further became orange-yellow product after stirring the reaction media for 7 min which indicated the existence of exocyclic *α*, *β*-unsaturated enone (**I**). Reaction progress was monitored via TLC technique and FTIR spectroscopy. Without any purification step, thiosemicarbazide (**3d**) was added in the same vessel which was refluxed in the presence of ethanol. The yield was improved by increasing the DEA concentration gradually from 1 to 1.3 mmol (Table 1, entry 7). Any further increase in DEA amount retarded the reaction due to the formation of imine as a side product. To avoid volatile organic solvents as much as possible and reduce reaction time, the above-mentioned reaction was also carried out in benign media such as neutral deep eutectic solvent (DES-1 to DES-3) and the results were compared with ethanol. It was observed that DES-1 produced an excellent yield (86 %) while DES-2 and DES-3 furnished lower yields and took comparatively longer time in product formation (Table 1, entry 12, 13, 14). It was envisioned to study the spiropyrazoline-indolinones synthesis at different temperatures, i.e., 25, 45, 65 and 70 °C using DES-1 which concluded 65 °C as the optimum reaction temperature as further increase in temperature decrease the yield (Table 1, entry 17). This could be explained by a lowering in H-bonding between the carbonyl oxygen of ketone and DES at elevated temperature.

**Table 1:** Optimization of reaction conditions under reflux.

Entry	Mole ratio of 1/2a/3d/	Base	Solvent	Temp (°C)	Time (min)	Yield (%)
**Selection of Base**						
1	1.0/1.0/1.0/1.0	KOH	Ethanol	75	180	45
2		NaOH			210	38
3		TEA			150	20
4		**DEA**			**90**	**65**
5		NH_3_			210	0
**Effect of Mole Ratio**						
6	1.0/1.0/1.0/1.2	DEA	Ethanol	75	90	42
7	**1.0/1.0/1.0/1.3**				**45**	**78**
8	1.0/1.0/1.0/1.4				120	72
9	1.0/1.0/1.0/1.5				180	65
10	1.0/1.0/1.0/3.0				180	35
**Effect of Solvents**						
11	1.0/1.0/1.0/1.3	DEA	Ethanol	75	45	78
12			**DES-1**		**24**	**81**
13			DES-2		65	68
14			DES-3		110	71
**Effect of Temperature**						
15	1.0/1.0/1.0/1.3	DEA	DES-1	25	180	0
16				45	150	20
17				**65**	**24**	**86**
18				70	30	79

All reactions were carried out by using 1 mmol of **1**, **2a** and **3d**. ^a^Isolated yields.

### 3.2 Microwave mediated synthesis of spiropyrazoline-indolinones (**4a–4t**)

Optimization studies were conducted to find the most suitable reaction conditions which were experimented to produce a variety of products in DES-1 using 5-Cl/Br isatin (**1a**/**1b**) with two different acetyl ketones (**2a** and **2b**) and different hydrazine derivatives (**3a–3e**) (Scheme 1). Targeted derivatives were produced after simple workup in 24 min to 3 h in a conventional setup (entries 7 and 9, Table 1). Hydrazine **3a** was employed to produce **4a,** it was formed in a longer time (2.5 h) with a low yield (42 %) while hydrazine **3b** gave a moderate yield (63 %) of **4b** in 1.5 h. Thiosemicarbazide (**3d**) furnished **4d** with a high yield (86 %) in 24 min which is due to the good nucleophilicity of hydrazine moiety while others are less reactive due to electron withdrawing functionalities (Table 3). The overall outcomes showing the reactivity of hydrazine analogs in the case of **1a** with **2a** are mentioned in descending order as **3d > 3e > 3b > 3c > 3a**. After examining the behavior of acetophenone (**2a**), the next 2-acetyl thiophene (**2b**) was reacted which showed the same reactivity order as observed in the case of ketone **2a**. This observation indicated that the electron withdrawing ability of chloro as well as nitro substituent on aromatic ring was responsible for decreasing the hydrazine reactivity. Overall **2a** produced better results than **2b** indicating the superiority of the phenyl ring of ketone over the thienyl ring. In the case of **1b** as substrate, hydrazine reactivity order was almost similar as mentioned above with slight variation. Hydrazine **3d** and **3e** reacted faster than **3c** when acetophenone was used.

Only a few reports highlight the clean and fast synthesis of spiropyrazoline in microwave [29]. So, inspired by this fact, an experimental study for new spiropyrazolines (**4a–4t**) has also been carried out at 70 ^ᵒ^C in the synthetic microwave.

To explore the optimum reaction conditions in a synthetic microwave reactor, a model reaction was performed using the equimolar ratio of the same reactants as used in classical settings. The reaction contents were irradiated at different power levels ranging from 100 to 280 W and product formation was observed by TLC. Results depicted that the reaction rate at 100 W was very slow which produced a 19% yield in 25 min whereas it was improved by increasing the power level gradually. At 240 W, the highest yield (82%) was obtained in 8 min (Table 2, entry 4), however, a further increase in power level furnished gummy product indicated impurities and side products. Once the best radiation level was achieved, the optimum catalyst amount was explored by using two concentrations of DEA in the reaction. At first, the equimolar ratio of reactants along with the catalyst was experimented, while 1.3 eq. of DEA was used in the second attempt (Table 2). Both the concentrations were applied by performing several reactions at different times. In the case of 1:1:1, 8 min was the optimum time for the required product (Table 2, entry 4). It was observed that an increase in reaction time decreased the yield. This could be rationalized by the high heating effect of the reaction chamber.

**Table 2:** Optimization of reaction conditions under microwaves.

Entry	Power level (W)	Mole ratio of 1/2a/3d	Time (min)	Yield (%)
1	100	1.0/1.0/1.0/1.0	25	19
2	150		25	39
3	200		18	45
4	**240**		**8**	**82**
5	280		10	0
6	240		15	70
7			18	66
8			8	33
9		1.0/1.0/1.0/1.3	15	34
10			18	52

In the case of ratio 1:1:1.3, it was investigated that comparable yields were recorded at 8 and 15 min whereas after 18 min irradiations maximum yield (52 %) was achieved. These observations concluded that the mole ratio 1:1:1 was better than 1:1:1.3 in terms of reaction time product yield. Versatility and the scope of the adopted procedure were studied by synthesizing a series of spiropyrazolines (**4a–4t**) from diketones. The mild reaction conditions enabled the incorporation of synthetically useful functionalities in the substrate. Its internal heating is more homogeneous than classical heating.

The effect of substituted hydrazines (**3a–e**) was explored by comparing the yield of the desired products and reaction time. The spiropyrazoline **4a** and **4b** were furnished using **3a** and **3b,** respectively, in 9 min with moderate yield (58 %) to good yield (75%), revealing the role of chloro group at phenyl moiety. The product **4c** was synthesized in 10 min with moderate yield due to the presence of electron-withdrawing effect of two nitro groups on the phenyl ring of hydrazine (**3c)**. The best results were obtained in 8 min for the formation of **4d** with 82% yield. The yield of **4e** was also close to that of **4d** and this observation is rationalized by the appreciable nucleophilic character of hydrazine **3d** and **3e**.

From the results, it was inferred that the reactivity of hydrazine **3b** was better than its analogs **3a a**nd **3c** when reacted with acetophenone. In the case of 2-acetyl thiophene, **3d** is the most reactive as compared to other analogs. However, **4l** was produced in 76 % in 12 min when reacted with **3b** (Table 3, entry 12). To study the effect of two diketones; **1a** and **1b**, the yield of **4a**-**4j** was compared with **4k**-**4t** and 5-bromo isatin was found more reactive. The effect of ketones **2a** and **2b** was also understandable by comparing yield which showed better results with 2-acetyl thiophene (**4f**-**4j**). Conventional and microwave approach of synthesis was analyzed for Scheme 1 which concluded that the classical method was lengthier in reaction time (0.5–3 h) in the presence of ethanol with moderate yield (41–86 %). While microwave-assisted synthesis (time 8–17 min, with moderate yield (41–86 %) is preferred; Microwave method > Conventional method. However, ethanol has been used as a reaction medium to facilitate microwave heating while conventional setup has used biodegradable deep eutectic solvent. It is easily prepared from non-toxic and low-cost chemicals. Their compositional flexibility makes it popular for the preparation of a variety of spiropyrazolines. From an environmental point of view, the classical synthesis described here is preferred over non-classical reaction setup (Table 3). If the microwave is not available, spiropyrazolines can be successfully prepared in DES, avoiding volatile organic solvents. Physical properties and spectroscopic data of newly synthesized spiropyrazoline-indolinones (**4a–4t**) are mentioned in supporting information.

**Table 3:** Conventional vs microwave method.

Entry	Compounds	Reactants	Conventional		Microwave	
			Time (hr)	Yield (%)	Time (min)	Yield (%)
1	**4a**	1a/2a/3a	2.5	42	9	58
2	**4b**	1a/2a/3b	1.5	63	9	75
3	**4c**	1a/2a/3c	2.5	65	10	70
4	**4d**	1a/2a/3d	**24 (min)**	**86**	**8**	**82**
5	**4e**	1a/2a/3e	1.5	77	17	80
6	**4f**	1a/2b/3a	2	41	12	60
7	**4g**	1a/2b/3b	2	62	11	74
8	**4h**	1a/2b/3c	3	52	8	63
9	**4i**	1a/2b/3d	2.5	68	10	90
10	**4j**	1b/2b/3e	2	58	11	88
11	**4k**	1b/2a/3a	1.5	68	14	57
12	**4l**	1b/2a/3b	3	62	12	76
13	**4m**	1b/2a/3c	3	68	10	74
14	**4n**	1b/2a/3d	0.5	82	9	90
15	**4o**	1b/2a/3e	1.5	75	11	79
16	**4p**	1b/2b/3a	2.5	58	13	73
17	**4q**	1b/2b/3b	3	64	12	82
18	**4r**	1b/2b/3c	2	70	10	81
19	**4s**	1b/2b/3d	1.5	78	11	89
20	**4t**	1b/2b/3e	3	74	10	84

All prepared DESs were confirmed by FTIR [30]. The freezing point of DES-1 [31], DES-2 [[32], [33], [34]] and DES-3 [35,36] was found to be 12. 5 and −40 °C respectively. Structure confirmation of newly synthesized compounds was achieved with elemental analysis, ^1^HNMR and ^13^CNMR and FTIR spectroscopy. The spectral data is well agreed with the literature values [37]. Based on the most supportive ^13^C NMR data, the spiro junction in spiropyrazoline was confidently assigned as a quaternary carbon at 70.21 ppm. The formation of diazole rings was confirmed by an absorption band in the FTIR spectrum at 1250 cm^−1^ for the C–N stretch and 3232 cm^−1^ for the N–H stretch. The ^1^H NMR spectrum confirmed this, with two singlets at 6.91 and 6.24 ppm assigned to H-4' and the hydrogen attached to the diazole nitrogen, respectively. Furthermore, in the ^1^H NMR spectrum, a set of doublets at 6.94 ppm and a double doublet at 7.38 ppm confirmed the involvement of the ketone skeleton provided by **1a**. The phenyl protons resonating at 7.52–7.64 ppm indicated that acetophenone had successfully contributed to ring formation, which was supported by the quaternary carbon resonating at 127.05 ppm (C-5 of **4a**). The FTIR spectrum provided additional confirmation, with absorption at 1608 cm^−1^ indicating the presence of a C=C stretch for the diazole ring. Aromatic protons attached to nitrogen were responsible for the multiplet observed in the ^1^H NMR spectrum at 7.71–7.89 ppm. In addition, the C=O group at C-2 was assigned a downfield signal in the ^13^C NMR spectrum at 162.84 ppm.

The ^1^HNMR spectra of **4b** to **4t**, revealed a singlet at 6.62–6.91 ppm designated to H-4. On the other hand, its ^13^CNMR was found to be supportive by displaying a quaternary carbon singlet in the range of 69.21–70.83 ppm as proof of spiro 1,2 diazole ring formation. In **4e**, an additional signal observed at 29.24 ppm indicated the (C-3′′′) of thiosemicarbazide in ^13^CNMR. Compound **4f** displayed a multiplet at 7.71–7.84 ppm, designated to thiophene moiety in the ^1^HNMR spectrum. The ^1^HNMR spectrum of **4g**, a multiplet (4H) resonated in the aromatic region 7.42–7.59 ppm was attributed to phenyl protons as a substituent of hydrazine. The ^1^HNMR spectrum of **4h**, showed a double doublet at 8.30 ppm and two doublets at 7.69 and 7.94 ppm with the ABX splitting pattern showing the presence of 2,4-dinitro phenyl moiety. The ^13^CNMR spectrum of **4i** revealed a high field signal at 177.36 ppm designated to thioamide moiety while a signal at 106.63 ppm was attributed to methine carbon as C-4′. Some of the significant signals in ^13^CNMR of **4j** exhibited at 166.46 ppm were credited to C-2 of 5-chloroisatin, 106.43 ppm to C-4′, 27.46 and 177.32 ppm were designated to methyl and quaternary carbon of thiosemicarbazide. The compounds from **4k** to **4t** are bromine analogs of spiropyrazoline and are in close agreement with chlorine analogs **4a** to **4j**. Nonetheless, condensations as well as cycloaddition reactions have a high potential to furnish very interesting spirocyclic frameworks. In the light of high demand of spiropyrazolines, it is speculated that the development of this new synthetic protocol will also present future insight into this area.

### 3.3 Computational studies

**Fig. 3:**
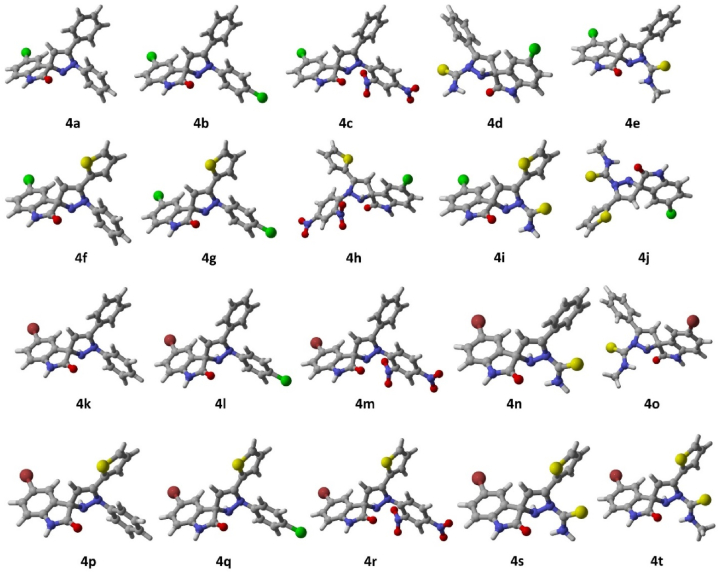
The optimized structures of chlorospiropyra acetophenones (**4a–4e**), chlorospiropyra thiophenes (**4f–4j**), bromospiropyra acetophenones (**4k–4o**), and bromospiropyra thiophenes (**4p–4t**) were obtained at the PBE0-D3BJ/def2-TZVP/SMD_DMSO_ level of theory. In the 3D models, the color scheme is as follows: grey represents carbon, white represents hydrogen, green corresponds to chlorine atoms, yellow represents sulfur, red denotes oxygen, brown is for bromine, and blue signifies nitrogen atoms.

**Fig. 4:**
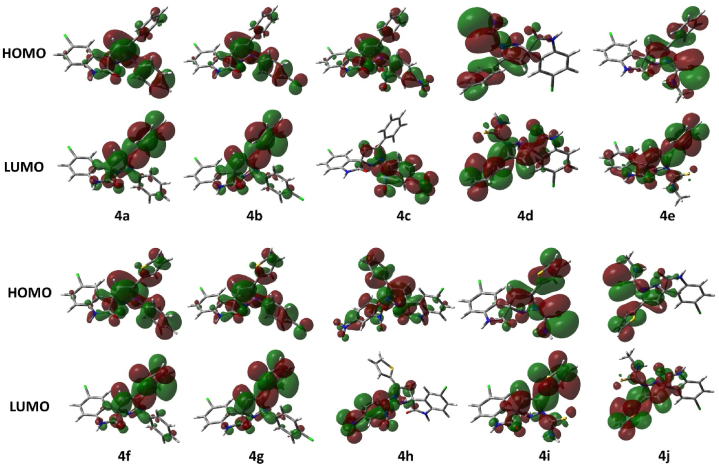
A presentation of the frontier orbitals of the chlorospiropyra acetophenones (**4a–4e**), chlorospiropyra thiophenes (**4f–4j**) calculated at PBE0-D3BJ/def2-TZVP/SMD_DMSO_ level of theory.

**Fig. 5:**
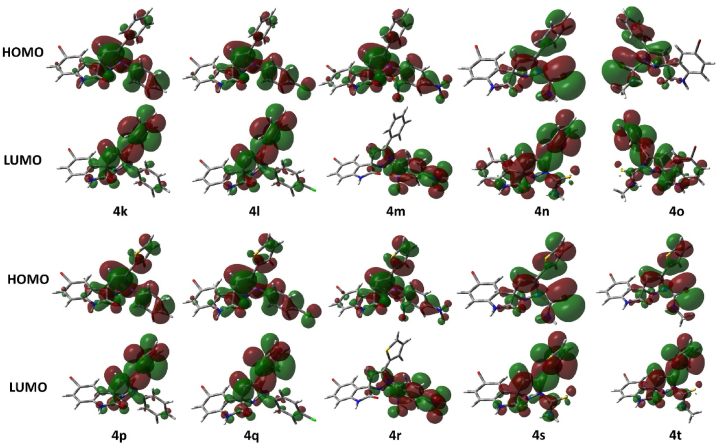
A presentation of the frontier orbitals of the bromospiropyra acetophenones (**4k–4o**), and bromospiropyra thiophenes (**4p–4t**) calculated at PBE0-D3BJ/def2-TZVP/SMD_DMSO_ level of theory.

Theoretical studies serve as a complement to verify and support experimental data. DFT serves as a powerful tool to predict various properties including chemical reactivity, stability and electronic structure. Gaussian 09 revision D.01 [38] has been used for all the calculations done in the present study. All the calculations have been performed using density functional theory (DFT) using the density functional PBE0 [39,40] with a triple ζ basis set def2-TZVP [41]. The non-bonding effects in the molecules were added using Grimme’s empirical dispersion correction [[42], [43], [44]] as implemented in Gaussian 09. The said method has been benchmarked earlier by us on a variety of similar studies and others as well and it proved to produce good results [[45], [46], [47], [48], [49], [50], [51]]. Solvent effects were added through the Polarizable Continuum Model (PCM) [[52], [53], [54], [55], [56], [57], [58]] added through Truhlar’s SMD parameter [59]. Frequency calculations on optimized geometries have been performed to confirm the structures as true minima by the absence of imaginary frequencies. GaussView and CYLview [60] software tools have been used to visualize the calculation results and produce figures for the manuscript. The optimized structures are depicted in Fig. 3(a–t), i.e., chlorospiropyra acetophenones (Fig. 3**4a–4e**), chlorospiropyra thiophenes (Fig. 3**4f–4j**), bromospiropyra acetophenones (Fig. 3**4k–4o**), and bromospiropyra thiophenes (Fig. 3**4p–4t**) were 3D modelled and optimized using Gaussian 09 rev. D01 software. The optimized structures were then analyzed for vibrational frequencies to confirm the absence of imaginary frequencies, establishing them as true potential energy surface (PES) minima. DFT calculations were used to determine the Frontier Molecular Orbital (FMO) Analysis, hyperpolarizability, molecular reactivity, and physical properties of compounds are computed and are depicted in Fig. 4(**a–j**), i.e., (**4a–4e**) the frontier orbitals of the chlorospiropyra acetophenones and (**4f–4j**) chlorospiropyra thiophenes. The frontier orbitals were calculated at PBE0-D3BJ/def2-TZVP/SMD_DMSO_ level of theory and are presented in Fig. 5(**a–t**), i.e., bromospiropyra acetophenones (**4k–4o**), and bromospiropyra thiophenes (**4p–4t**) [61]**.**

Table 4 enlists the HOMO-LUMO gap (ΔE) and hyperpolarizability (*β*) values of all the compounds (**4a–4t**). The ΔE values of these molecules are in a relatively narrow range from 3.63 to 4.79 eV which means that their reactivity is almost similar. The lowest ΔE value of compound **4h** (3.63 eV) suggests it to be the most reactive among the series. That can be attributed to the inductive electron withdrawing –Cl group on one phenyl ring and –NO_2_ group on the other phenyl ring in the molecule. Similarly, **4d** has the highest ΔE value (4.79 eV) which establishes it to be the most stable in the series. The next most stable compounds are **4k** and **4l** which have deactivating Br group on the aromatic ring. All the HOMO and LUMO energies are given in eV.

**Table 4:** HOMO-LUMO energies and gap (expressed in eV) of the compounds **4a–4t**.

Compound	E_*HOMO*_	E_*LUMO*_	HOMO-LUMO Gap	Hyperpolarizability
**4a**	−5.77	−1.14	4.63	544.40
**4b**	−5.77	−1.16	4.61	193.84
**4c**	−6.15	−2.53	3.63	9970.51
**4d**	−5.93	−1.14	4.79	718.30
**4e**	−5.87	−1.11	4.76	785.24
**4f**	−5.82	−1.30	4.52	672.62
**4g**	−5.83	−1.34	4.48	549.75
**4h**	−6.18	−2.54	3.63	10118.86
**4i**	−5.82	−1.26	4.56	763.98
**4j**	−5.76	−1.24	4.53	749.80
**4k**	−5.78	−1.14	4.64	382.26
**4l**	−5.78	−1.17	4.62	221.69
**4m**	−6.18	−2.53	3.65	11141.77
**4n**	−5.93	−1.14	4.78	886.16
**4o**	−5.88	−1.12	4.76	946.03
**4p**	−5.83	−1.30	4.53	599.68
**4q**	−5.83	−1.33	4.50	585.29
**4r**	−6.18	−2.55	3.63	10020.02
**4s**	−5.82	−1.26	4.55	911.93
**4t**	−5.77	−1.25	4.52	918.55

The distribution of iso-density seems very similar in these compounds. In chlorospiropyra acetophenones (**4a–4e**), chlorospiropyra thiophenes (**4f–4j**), bromospiropyra acetophenones (**4k–4o**), and bromospiropyra thiophenes (**4p–4t**), the iso-density is mainly spread over the whole molecule except the chloro- and bromo-substituted rings in all the compounds under study for HOMO. Interestingly, LUMO iso-density is also located on the rest of the molecule except the chloro and bromo-substituted aromatic rings. Only in the case of compound **4o**, it is located on the bromophenyl ring as well to some extent. The hyperpolarizability (*β*) values of the compounds under study do not show them as potent non-linear optical (NLO) materials but four of them (**4c**, **4h**, **4m**, and **4r**) show quite good NLO response. The highest *β* value is shown by compound **4m** and the second highest is shown by **4c**, **4h** and **4r**. That can be explained by the presence of the –NO_2_ group as a deactivator and the bromide as a weak activator on the other side in compound **4m**. These groups govern the electron’s push and pull mechanism in these molecules.

#### 3.3.1 Molecular electrostatic potential

**Fig. 6:**
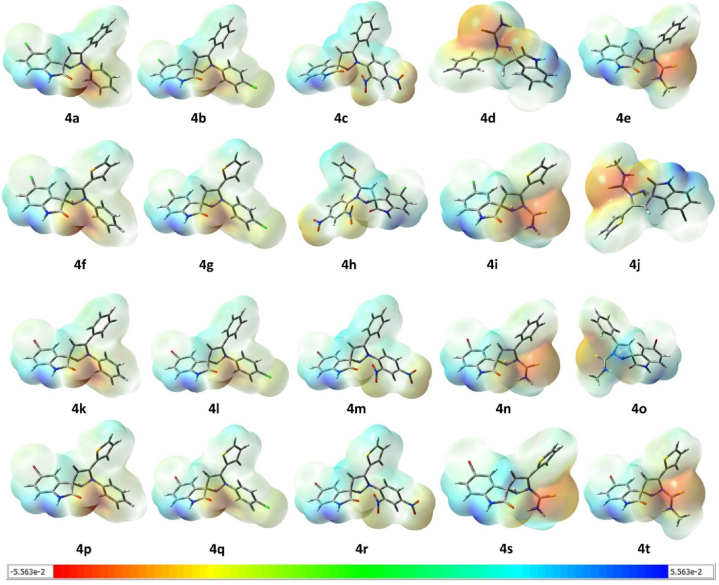
A presentation of all the molecular electrostatic potentials of the molecules (**4a–4t**) calculated at PBE0-D3BJ/def2-TZVP/SMD_DMSO_ level of theory. The scale below shows the colour coding. The values in the scale are in Hartrees.

MEP maps (molecular electrostatic potential) are useful 3D plots for visualizing the charge distribution, size, and shape of compounds under investigation. These maps depict a proton's energy about its current position. The density of electrons is represented in different colors on these maps, providing information about a molecule relative polarity. The molecular electrostatic potentials of the molecules are presented in Fig. 6(**4a–4t**) calculated at PBE0-D3BJ/def2-TZVP/SMD_DMSO_ level of theory. In Fig. 6(**4a–4t**), the electron-rich nucleophilic positions of the molecule in red, while sites with lower electron density are depicted in blue can be seen. So, it can be understood in terms of nucleophilic or electrophilic sites, Fig. 6(**4a–4t**) can be visualized individually for their electrophilic and nucleophilic sites. In compounds **4h**, **4m**, and **4r** the electron density is distributed equally throughout the molecule while in other compounds the red color depicts the unequal charge distribution on the molecule.

#### 3.3.2 Conceptual DFT reactivity descriptors

The chemical reactivity descriptors of a molecule include electron affinity (*A*), ionization potential (*I*), chemical hardness (*ƞ*), electronic chemical potential (*μ*), and Nucleophilicity Index (*N*) which were determined by FMO calculations. The values of all the important reactivity descriptors of the compounds under study are given in Table 5. The Koopman’s theorem was used to calculate *I* and *A* which stated that the negative of E_*HOMO*_ and E_*LUMO*_ correspond to the ionization potential (*I*) and electron affinity (*A*) of the compound [62,63]. The other descriptors i.e., *η* (chemical hardness), *μ* (electronic chemical potential), ω (electrophilicity index), and *N* (Nucleophilicity Index) are then subsequently calculated as shown in Eqs. (1)–(4).

(1) $$\eta =\left(E_{HOMO}-E_{LUMO}\right)/2$$

(2) $$\mu =-\left(E_{HOMO}+E_{LUMO}\right)/2$$

(3) $$\omega =\mu^{2}/2\eta$$

(4) $$N=E_{\text{HOMO}\left(\text{Nu}\right)}\;–\;E_{\text{HOMO}\left(\text{TCE}\right)}$$

**Table 5:** Chemical reactivity descriptors of **4a–4t**.

Compound	Ionization Potential, *I* (eV)	Electron Affinity, *A* (eV)	Chemical hardness, *ƞ* (eV)	Electronic chemical potential, *μ* (eV)	Electrophilicity Index, *ω* (eV)	Nucleophilicity Index (*N*) eV
**4a**	5.77	1.14	−2.31	3.46	−2.58	3.349
**4b**	5.77	1.16	−2.31	3.47	−2.61	3.344
**4c**	6.15	2.53	−1.81	4.34	−5.20	2.965
**4d**	5.93	1.14	−2.39	3.53	−2.60	3.193
**4e**	5.87	1.11	−2.38	3.49	−2.56	3.246
**4f**	5.82	1.30	−2.26	3.56	−2.80	3.302
**4g**	5.83	1.34	−2.24	3.58	−2.86	3.292
**4h**	6.18	2.54	−1.82	4.36	−5.23	2.942
**4i**	5.82	1.26	−2.28	3.54	−2.74	3.303
**4j**	5.76	1.24	−2.26	3.50	−2.70	3.354
**4k**	5.78	1.14	−2.32	3.46	−2.59	3.337
**4l**	5.78	1.17	−2.31	3.48	−2.62	3.335
**4m**	6.18	2.53	−1.82	4.35	−5.19	2.940
**4n**	5.93	1.14	−2.39	3.53	−2.61	3.193
**4o**	5.88	1.12	−2.38	3.50	−2.58	3.240
**4p**	5.83	1.30	−2.27	3.56	−2.80	3.289
**4q**	5.83	1.33	−2.25	3.58	−2.85	3.288
**4r**	6.18	2.55	−1.81	4.36	−5.24	2.943
**4s**	5.82	1.26	−2.28	3.54	−2.75	3.303
**4t**	5.77	1.25	−2.26	3.51	−2.73	3.347

Table 5 shows the values of all the crucial reactivity descriptors for the compounds under investigation. Chemical hardness, a measure of a substance's resistance to deformation, is prominently featured. As the chemical hardness value increases, reactivity tends to decrease, indicating greater stability for the substance. So, it can be suggested that the HOMO-LUMO gap is supported by the *ƞ* values as compound **4h** has one of the lowest *ƞ* values and **4d** has the highest *ƞ* value. Chemical potential is the ability of a system to accept or donate the electrons, lower the chemical potential, lower the electron-acceptance and vice versa. In the light of chemical potential, **4h** shows highest *μ* value, supporting its electron-donating nature. Ionization potential and electron affinity values also supports the higher reactivity of **4h** as observed through chemical potential and electrophilicity index. The nucleophilicity index (*N*) is the measure of the ease of donation of an electron pair by a molecule or an atom to an electrophile and calculated, based on the scale proposed by Domingo [64]. Thus, higher the value of (*N*), higher is the reactivity towards electrophiles. According to Table 5, **4j** shows higher reactivity towards electrophiles and **4m** indicates lower reactivity towards the electrophiles.

## 4 Conclusion

In summary, highly functionalized new spiropyrazoline-indolinones have been developed through a facile, one-pot two-step, multicomponent green methodology. This protocol features catalyst-free conditions, green solvents, mild reaction conditions, easy work-up with high yield, and short reaction time. The tricyclic spiro framework, as the final product, could serve as important scaffolds for drug discovery and be advantageous for both medicinal and synthetic chemists. The structural and electronic properties can be understood through density functional theory calculations performed on the synthesized chlorospiropyra acetophenones (4a-4e) and thiophenes (4f-4j), as well as bromospiropyra acetophenones (4k-4o) and thiophenes (4p-4t). Furthermore, an examination of frontier orbitals and other reactivity descriptors, such as ionization potential, electron affinity, chemical hardness, electronic chemical potential, and electrophilicity index, revealed that compound 4h is the most reactive in the series, while compound 4d is the most stable.

## Research funding

This research was funded by Princess Nourah bint Abdulrahman University Researchers Supporting Project number (PNURSP2024R158), 10.13039/501100004242Princess Nourah bint Abdulrahman University, Riyadh, Saudi Arabia. The authors extend their appreciation to the Deanship of Scientific Research at 10.13039/501100007446King Khalid University for funding this work through large group Research Project under grant number RGP.2/575/44.

## Data availability statement

Data included in article/supp. material/referenced in article.

## CRediT authorship contribution statement

**Zubi Sadiq:** Writing – original draft, Investigation. **Ambreen Ghani:** Formal analysis, Data curation. **Muhammad A. Hashmi:** Software, Methodology, Data curation. **A. Dahshan:** Software, Formal analysis, Funding acquisition. **Shahnaz:** Project administration, Methodology. **Samiah H. Al-Mijalli:** Resources, Funding acquisition. **Munawar Iqbal:** Writing – review & editing, Validation. **Erum A. Hussain:** Methodology, Conceptualization.

## Declaration of competing interest

The authors declare that they have no known competing financial interests or personal relationships that could have appeared to influence the work reported in this paper.
