# Supplementary material for: Green synthesis of novel spiropyrazoline-indolinones in neutral deep eutectic solvents and DFT studies
Source: Heliyon. 2023 Dec 20;10(1):e23814. doi: 10.1016/j.heliyon.2023.e23814 (PMC10788502; doi:10.1016/j.heliyon.2023.e23814)
Supplement: Multimedia component 1 [file mmc1.docx]

**Supplementary material**

**Green Synthesis of Novel Spiropyrazoline-indolinones in Neutral Deep Eutectic Solvents and DFT Studies**

Zubi Sadiq^1^, Ambreen Ghani^2^, Muhammad A. Hashmi^3^, A. Dahshan^1,4^, Shahnaz^1^, Samiah H. Al-Mijalli^1,5^, Munawar Iqbal^6,*^ and Erum A. Hussain^1^

^1^Department of Chemistry, Lahore College for Women University, Lahore 54000, Pakistan

^2^Department of Chemistry, University of Education Lahore, Vehari Campus, 61100, Pakistan

^3^Department of Chemistry, University of Education, Attock Campus, Attock 43600, Pakistan

^4^Department of Physics - Faculty of Science - King Khalid University, P.O. Box 9004, Abha, Saudi Arabia

^5^Department of Biology, College of Sciences, Princess Nourah bint Abdulrahman University, P.O. Box 84428, Riyadh 11671, Saudi Arabia

^6^Department of Chemistry, Division of Science and Technology, University of Education, Lahore, Pakistan

*Correspondence author E-mail: [bosalvee@yahoo.com](mailto:bosalvee@yahoo.com)

**1.** **Physical properties and spectroscopic data of newly synthesized spiropyrazoline-indolinones (4a-4t)**

**1.1 5-Chloro-1',5'-diphenyl-1',2'-dihydrospiro[indoline-3,3'-pyrazol]-2-one (4a)** Mustard solid, C_22_H_16_ClN_3_O, 373 g/mol, R*_f_* 0.94, yield 650 mg, 58 %, Mp 213 ֯C. FTIR cm^-1^: 565 (C‒Cl), 1250 (C‒N), 1608 (C=C), 1697 (C=O), 3215 (N‒H), 3264 (N‒H isatin). ^1^HNMR (400 MHz, DMSO-*d*_6_) 6.24 (s, 1H, NH-pyra), 6.91 (s, 1H, H-4´), 6.94 (d, *J* = 8.32, 1H, H-7), 7.38 (dd, *J* = 8.31, 2.43, 1H, H-6), 7.52 - 7.64 (m, 5H Ar-Ph), 7.71-7.89 (m, 5H Ar-Ph), 7.94 (d, *J* = 2.44, 1H, H-4), 10.08 (s, 1H, NH). ^13^CNMR (75 MHz, DMSO-*d*_6_): 70.21 (C-3 spiro), 104.2 (C-4´), 113.65 (C-7), 121.13 (C-4´´´), 122.18 (C-3a), 122.34 (C-4), 123.04 (C-1´´´), 125.74 (C-3´´, C-5´´), 127.05 (C-5), 127.28 (C-4´´), 127.86 (C-2´´´, 6´´´), 128.35 (C-2´´, C-6´´), 128.71 (C-3´´´, C-5´´´), 131.96 (C-6), 132.02 (C-7a), 135.12 (C-1´´), 150.5 (C-5´), 162.84 (C-2). Anal. calculated (%) For: C 70.68; H 4.31; N 11.24 Found: C 70.65; H 4.30; N 11.21.

**1.2 5-Chloro-1'-(4-chlorophenyl)-5'-phenyl-1',2'-dihydrospiro[indoline-3,3'-pyrazol]-2-one (4b)** Orange red solid, C_22_H_15_Cl_2_N_3_O, 407 g/mol, R*_f_* 0.81, yield 915 mg, 75 %, Mp 215 ֯C. FTIR cm^-1^ : 568 (C‒Cl), 1252 (C‒N), 1606 (C=C), 1697 (C=O), 3220 (N‒H spiro), 3264 (N‒H isatin).^1^HNMR (400 MHz, DMSO-*d*_6_) 6.24 (s, 1H, NH-pyra). 6.83 (s, 1H, H-4´), 6.94 (d, *J* = 8.32, IH, H-7), 7.38 (dd, *J* = 8.31, 2.43, 1H, H-6), 7.46-7.58 (m, 5H Ar-Ph), 7.64-7.79 (m, 4H Ar-Ph), 7.83(d, *J* = 2.44, 1H, H-4), 10.06 (s, 1H, NH).^13^CNMR (75 MHz, DMSO-*d*_6_): 70.72 (C-3 spiro), 107.35 (C-4´), 114.67 (C-7), 123.18 (C-3a), 125.34 (C-4), 127.05 (C-5), 127.28 (C-4´´´), 127.89 (C-2´´´, 6´´´), 128.96 (C-3´´´, 5´´´), 129.02 (C-1´´´), 129.99 (C-4´´), 132.23 (C-7a), 132.45 (C-2´´, C-6´´), 133.96 (C-6), 135.28 (C-1´´), 138.74 (C-3´´, C-5´´), 150.5 (C-5´), 162.84 (C-2). Anal. calculated (%) For: C 64.72; H 3.70; N 10.29, Found: C 64.70; H 3.71; N 10.26.

**1.3 5-Chloro-1'-(2,4-dinitrophenyl)-5'-phenyl-1',2'-dihydrospiro[indoline-3,3'-pyrazol]-2-one (4c)**  Brick red solid, C_22_H_14_ClN_5_O_5_, 463 g/mol, R*_f_* 0.85 yield 973 mg, 70 %, Mp 216 ֯C. FTIR cm^-1^ : 566 (C‒Cl), 1254 (C‒N), 1604 (C=C), 1697 (C=O), 3218 (N‒H spiro), 3264 (N‒H isatin).^1^HNMR (400 MHz, DMSO-*d*_6_) 6.21 (s, 1H, NH-pyra), 6.62 (s, 1H, H-4´), 6.94 (d, *J* = 8.4, 1H, H-7), 7.41 (dd, *J* = 8.4, 2.43, 1H, H-6), 7.52-7.67 (m, 5H Ar-Ph), 7.78 (1H, d, *J* = 2.4 Hz, H-3 DNPh) 7.87 (d, 2.44, 1H, H-4), 7.96 (1H, d, 𝐽 = 8.6 Hz, H-6 DNPh), 8.35 (1H, dd, *J* = 8.6, 2.4 Hz, H-5 DNPh), 10.03(s, 1H, NH).^13^CNMR (75 MHz, DMSO-*d*_6_) 72.21 (C-3 spiro), 107.2 (C-2´), 115.65 (C-7), 122.13 (C-4´´´), 125.18 (C-3a), 126.34 (C-4), 127.05 (C-5), 127.86 (C-2´´´, 6´´´), 128.71 (C-3´´´, 5´´´), 129.04 (C-1´´´), 129.35 (C-2´´), 131.96 (C-6), 132.02 (C-7a), 132.27 (C-4´´), 135.28 (C-1´´), 136.57 (C-5´´), 137.87 (C-6´´), 139.74 (C-3´´), 151.45 (C-1´), 164.84 (C-2). Anal. calculated (%) For: C 56.97; H 3.04; N 15.10, Found: C 56.95; H 3.01; N 15.08.

**1.4 5-Chloro-2-oxo-5'-phenylspiro[indoline-3,3'-pyrazole]-1'(2'*H*)-carbothioamide (4d)** Yellow solid, C_17_H_13_ClN_4_OS, 356 g/mol, R*_f_* 0.97, Yield 876 mg, 82 %, Mp 205 ֯C. FTIR cm^-1^ : 569 (C‒Cl), 1226 (C=S), 1256 (C‒N), 1609 (C=C), 1697 (C=O), 3221 (N‒H spiro), 3264 (N‒H isatin).^1^HNMR (400 MHz, DMSO-*d*_6_) 6.22 (s, 1H, NH-pyra), 6.83 (s, 1H, H-4´), 6.94 (d, *J* = 8.32, 1H, H-7), 7.38 (dd, *J* = 8.31, 2.43, 1H, H-6), 7.52-7.64 (m, 5H Ar-Ph), 7.84 (d, *J* = 2.44, 1H, H-4), 10.06 (s, 1H, NH).^13^CNMR (75 MHz, DMSO-*d*_6_) 69.21 (C-3 spiro), 106.2 (C-4´), 114.65 (C-7), 122.13 (C-4´´´), 124.18 (C-3a), 124.34 (C-4), 126.04 (C-1´´´), 128.05 (C-5), 129.71 (C-3´´´, 5´´´), 129.86 (C-2´´´, 6´´´), 131.96 (C-6), 133.02 (C-7a), 151.25 (C-5´), 165.79 (C-2), 177.36 (C-1´´). Anal. calculated (%) For: C 57.22; H 3.39; N 15.70; S 8.99, Found: C 57.19; H 3.36; N 15.68; S 8.94.

**1.5 5-Chloro-N-methyl-2-oxo-5'-phenylspiro[indoline-3,3'-pyrazole]-1'(2'*H*)-carbothioamide (4e)** Pale yellow solid C_18_H_15_ClN_4_OS, 370 g/mol, R*_f_* 0.92, Yield 888 mg, 80 %, Mp 207 ֯C. FTIR cm^-1^ : 1697 (C=O), 3264 (N‒H isatin), 563 (C‒Cl), 3222 (N‒H spiro), 1250 (C‒N), 1606 (C=C), 1224 (C=S).^1^HNMR (400 MHz, DMSO-*d*_6_) 2.57 (s, 3H), 6.22 (s, 1H, NH-pyra), 6.83 (s, 1H, H-4´), 6.94 (d, *J* = 8.32, 1H, H-7), 7.38 (dd, *J* = 8.31, 2.43, 1H, H-6), 7.51 - 7.66 (m, 5H Ar-Ph), 7.86 (d, *J* = 2.44, 1H, H-4), 10.08 (s, 1H, NH).^13^CNMR (75 MHz, DMSO-*d*_6_) 29.24 (C-3´´´), 69.21(C-3 spiro), 106.2 (C-4´), 114.65 (C-7), 122.13 (C-4´´´), 124.18 (C-3a), 124.34 (C-4), 128.05 (C-5), 129.71 (C-3´´´, 5´´´), 129.86 (C-2´´´, 6´´´), 131.96 (C-6), 133.02 (C-7a), 151.25 (C-5´), 165.79 (C-2), 171.36 (C-1´´´). Anal. calculated (%) For: C 58.30; H 4.08; N 15.11; S 8.65, Found: C 58.29; H 4.04; N 15.09; S 8.63.

**1.6 5-Chloro-1'-phenyl-5'-(thiophen-2-yl)-1',2'-dihydrospiro[indoline-3,3'-pyrazol]-2-one (4f)** Brown solid, C_20_H_14_ClN_3_OS, 379 g/mol, R*_f_* 0.77 Yield 682 mg, 60 %, Mp 215 ֯C. FTIR cm^-1^ : 1697 (C=O), 3264 (N‒H isatin), 569 (C‒Cl), 3220 (N‒H spiro), 1254 (C‒N), 1609 (C=C) 704 (C‒S).^1^HNMR (400 MHz, DMSO-*d*_6_) 6.24 (s, 1H, NH-pyra), 6.91 (s, 1H, H-4´), 6.94 (d, *J* = 8.32, 1H, H-7), 7.38 (dd, *J* = 8.31, 2.43, 1H, H-6), 7.54 - 7.67 (m, 5H Ar-Ph), 7.71-7.89 (m, 3H Ar-Ph), 7.94 (d, *J* = 2.44, 1H, H-4), 10.08 (s, 1H, NH).^13^CNMR (75 MHz, DMSO-*d*_6_) 70.21 (C-3 spiro), 104.2 (C-4´), 113.65 (C-7), 122.18 (C-3a), 122.34 (C-4), 125.74 (C-3´´, C-5´´), 127.05 (C-5), 127.28 (C-4´´), 127.86 (C-2´´´), 128.35 (C-2´´, C-6´´), 128.71 (C-3´´´), 131.96 (C-6), 132.02 (C-7a), 135.12 (C-1´´), 142.04 (C-1´´´), 147.62 (C-4´´´), 151.75 (C-5´), 162.84 (C-2). Anal. calculated (%) For: C 63.24; H 3.72; N 11.06; S 8.44, Found: C 63.21; H 3.70; N 11.03; S 8.42.

**1.7 5-Chloro-1'-(4-chlorophenyl)-5'-(thiophen-2-yl)-1',2'-dihydrospiro[indoline-3,3'-pyrazol]-2-one (4g)** Orange solid, C_20_H_13_Cl_2_N_3_OS, 413 g/mol, R*_f_* 0.82 yield 917 mg, 74 %, Mp 220 ֯C. FTIR cm^-1^ : 1697 (C=O), 3264 (N‒H isatin), 567 (C‒Cl), 3220 (N‒H spiro), 1252 (C‒N), 1606 (C=C), 706 (C‒S).^1^HNMR (400 MHz, DMSO-*d*_6_) 6.24 (s, 1H, NH-pyra), 6.83 (s, 1H, H-4´), 6.94 (d, *J* = 8.32, 1H, H-7), 7.38 (dd, *J* = 8.31, 2.43, 1H, H-6), 7.42-7.59 (m, 4H Ar-Ph), 7.64 (m, 3H Ar-Ph), 7.83(d, *J* = 2.44, 1H, H-4), 10.08 (s, 1H, NH).^13^CNMR (75 MHz, DMSO-*d*_6_) 70.37 (C-3 spiro), 106.2 (C-4´), 113.65 (C-7), 122.18 (C-3a), 122.34 (C-4), 127.05 (C-5), 128.71 (C-3´´´), 131.35 (C-2´´, C-6´´), 131.96 (C-6), 132.02 (C-7a), 133.28 (C-4´´), 135.2 (C-1´´),135.86 (C-2´´´), 129.13 (C-4´´´), 138.74 (C-3´´, C-5´´), 142.0 (C-1´´´), 150.5 (C-5´), 162.84 (C-2). Anal. calculated (%) For: C 57.98; H 3.16; N 10.14; S 7.74, Found: C 57.95; H 3.14; N 10.11; S 7.72.

**1.8 5-Chloro-1'-(2,4-dinitrophenyl)-5'-(thiophen-2-yl)-1',2'-dihydrospiro[indoline-3,3'-pyrazol]-2-one (4h),**  Brick red solid, C_20_H_12_ClN_5_O_5_S, 469 g/mol, R*_f_* 0.87 yield 882 mg, 63 %, Mp 222 ֯C. FTIR cm^-1^ : 1697 (C=O), 3264 (N‒H isatin), 568 (C‒Cl), 3224 (N‒H spiro), 1258 (C‒N), 1607 (C=C), 704 (C‒S).^1^HNMR (400 MHz, DMSO-*d*_6_) 6.83 (s, 1H, H-4´), 6.91 (s, 1H, NH pyra), 6.94 (d, *J* = 8.32, 1H, H-7), 7.38 (dd, *J* = 8.31, 2.43, 1H, H-6), 7.55 (1H, dd, *J* = 8.8 Hz, H-3 DNPh), 7.69 (1H, d, *J* = 8.8 Hz, H-6 DNPh), 7.71-7.89 (m, 3H Ar-Ph), 7.94 (d, *J* = 2.44, 1H, H-4), 8.30 (1H, dd, *J* = 8.8, 2.3 Hz, H-5DNPh), 10.08 (s, 1H, NH).^13^CNMR (75 MHz, DMSO-*d*_6_) 72.21 (C-3 spiro), 107.2 (C-2´), 115.65 (C-7), 125.18 (C-3a), 126.34 (C-4), 127.05 (C-5), 127.86 (C-4´´´), 128.71 (C-3´´´), 129.35 (C-2´´), 131.73(C-5´´´), 131.96 (C-6), 132.02 (C-7a), 132.27 (C-4´´), 135.28 (C-1´´), 136.57 (C-5´´), 137.87 (C-6´´), 139.74 (C-3´´), 142.04 (C-2´´´), 151.45 (C-1´), 164.84 (C-2). Anal. calculated (%) For: C 51.13; H 2.57; N 14.91; S 6.82 Found: C 51.11; H 2.54; N 14.89; S 6.80.

**1.9 5-Chloro-2-oxo-5'-(thiophen-2-yl)spiro[indoline-3,3'-pyrazole]-1'(*2'H*)-carbothioamide (4i)** Orange solid, C_15_H_11_ClN_4_OS_2_ 362 g/mol, R*_f_* 0.90, yield 978 mg, 90 %, Mp 210 ֯C. FTIR cm^-1^ : 1697 (C=O), 3264 (N‒H isatin), 565 (C‒Cl), 3219 (N‒H spiro), 1256 (C‒N), 1604 (C=C), 708 (C‒S), 1232 (C=S). ^1^HNMR (400 MHz, DMSO-*d*_6_) 6.22 (s, 1H, NH-pyra), 6.83 (s, 1H, H-4´), 6.94 (d, *J* = 8.32, 1H, H-7), 7.38 (dd, *J* = 8.31, 2.43, 1H, H-6), 7.52-7.64 (m, 3H Ar-Ph), 7.84 (d, *J =* 2.44, 1H, H-4), 10.08 (s, 1H, NH). ^13^CNMR (75 MHz, DMSO-*d*_6_) 69.42 (C-3 spiro), 106.63 (C-4´), 114.87 (C-7), 122.53 (C-4´´´), 124.24 (C-3a), 124.38 (C-4), 126.25 (C-1´´´), 128.13 (C-5), 129.58 (C-3´´´, 5´´´), 129.97 (C-2´´´, 6´´´), 132.06 (C-6), 133.23 (C-7a), 151.28 (C-5´), 166.46 (C-2), 177.36 (C-1´´). Anal. calculated (%) For: C 49.65; H 3.06; N 15.44; S 17.67, Found: C 49.63; H 3.02; N 15.43; S 17.69.

**1.10 5-Chloro-N-methyl-2-oxo-5'-(thiophen-2-yl)spiro[indoline-3,3'-pyrazole]-1'(2*'H*)-carbothioamide (4j)** Orange solid, C_16_H_13_ClN_4_OS_2_ 376 g/mol, R*_f_* 0.64 yield 993 mg, 88 %, Mp 212 ֯C. FTIR cm^-1^: 1697 (C=O), 3264 (N‒H isatin), 564 (C‒Cl), 3223 (N‒H spiro), 1252 (C‒N), 1609 (C=C), 706 (C‒S), 1226 (C=S). ^1^HNMR (400 MHz, DMSO-*d*_6_) 2.64 (s, 3H), 6.18 (s, 1H, NH-pyra), 6.78 (s, 1H, H-4´), 6.91 (d, *J* = 8.4, 1H, H-7), 7.39 (dd, *J* = 8.4, 2.2, 1H, H-6), 7.49-7.62 (m, 3H Ar-Ph), 7.84 (d, *J* = 2.2, 1H, H-4), 10.08 (s, 1H, NH). ^13^CNMR (75 MHz, DMSO-*d*_6_) 27.46 (C-3´´), 69.42(C-3 spiro), 106.63 (C-4´), 114.87 (C-7), 122.53 (C-4´´´), 124.24 (C-3a), 124.38 (C-4), 126.25 (C-5´´´), 128.13 (C-5), 129.58 (C-3´´´), 129.97 (C-2´´´), 132.06 (C-6), 133.23 (C-7a), 151.28 (C-5´), 166.46 (C-2), 177.32 (C-1´´). Anal. calculated (%) For: C 50.99; H 3.48; N 14.87; S 17.01, Found: C 50.96; H 3.47; N 14.85; S 17.04.

**1.11 5-Bromo-1',5'-diphenyl-1',2'-dihydrospiro[indoline-3,3'-pyrazol]-2-one (4k)** Yellow orange solid, C_22_H_16_BrN_3_O, 418 g/mol, R*_f_* 0.94, yield 712 mg, 57 %, Mp 218 ֯C. FTIR cm^-1^ : 1697 (C=O), 3264 (N‒H isatin), 665 (C‒Br), 3218 (N‒H spiro), 1268 (C‒N), 1607 (C=C). ^1^HNMR (400 MHz, DMSO-*d*_6_) 6.24 (s, 1H, NH-pyra), 6.91 (s, 1H, H-4´), 6.94 (d, *J* = 8.32, 1H, H-7), 7.42 (dd, *J* = 8.31, 2.43, 1H, H-6), 7.52-7.64 (m, 5H Ar-Ph), 7.71-7.89 (m, 5H Ar-Ph), 7.91 (d, *J* = 2.44, 1H, H-4), 10.14 (s, 1H, NH). ^13^CNMR (75 MHz, DMSO-*d*_6_) 70.21 (C-3 spiro), 103.29 (C-4´), 115.85 (C-7), 122.35 (C-4´´´), 123.14 (C-3a), 123.38 (C-4), 124.06 (C-1´´´), 125.84 (C-3´´, C-5´´), 127.12 (C-5), 127.45 (C-4´´), 127.97 (C-2´´´, C-6´´´), 128.39 (C-2´´, C-6´´), 128.76 (C-3´´´, C-5´´´), 129.73 (C-4´´´), 132.06 (C-6), 132.15 (C-7a), 135.26 (C-1´´), 151.85 (C-5´), 163.84 (C-2). Anal. calculated (%) For: C 63.17; H 3.86; N 10.05, Found: C 63.15; H 3.83; N 10.02.

**1.12 5-Bromo-1'-(4-chlorophenyl)-5'-phenyl-1',2'-dihydrospiro[indoline-3,3'-pyrazol]-2-one (4l)** Orange solid, C_22_H_15_BrClN_3_O, 452 g/mol, R*_f_* 0.78, yield 1026 mg, 76 %, Mp 222 ֯C. FTIR cm^-1^: 1697 (C=O), 3264 (N‒H isatin), 669 (C‒Br), 3221 (N‒H spiro), 1266 (C‒N), 1605 (C=C). ^1^HNMR (400 MHz, DMSO-*d*_6_): 6.24 (s, 1H, NH-pyra), 6.83 (s, 1H, H-4´), 6.94 (d, *J* = 8.32, 1H, H-7), 7.38 (dd, *J* = 8.31, 2.43, 1H, H-6), 7.52-7.67 (m, 4H Ar-Ph), 7.73-7.86 (m, 5H Ar-Ph), 7.87 (d, *J* = 2.44, 1H, H-4), 10.12 (s, 1H, NH). ^13^CNMR (75 MHz, DMSO-*d*_6_): 70.81 (C-3 spiro), 108.43 (C-4´), 115.74 (C-7), 123.18 (C-3a), 124.34 (C-4), 125.37 (C-1´), 127.25 (C-5), 127.46 (C-4´´´), 128.08 (C-2´´´, 6´´´), 128.84 (C-3´´´, 5´´´), 129.96 (C- 4´´), 132.63 (C-7a), 133.97 (C-2´´, C-6´´), 134.04 (C-6), 135.47 (C-1´´), 139.35 (C-3´´, C-5´´), 152.85 (C-5´), 161.92 (C-2). Anal. calculated (%) For: C 58.37; H 3.34; N 9.28, Found: C 58.35; H 3.33; N 9.25.

**1.13 5-Bromo-1'-(2,4-dinitrophenyl)-5'-phenyl-1',2'-dihydrospiro[indoline-3,3'-pyrazol]-2-one (4m)** Orange red solid, C_22_H_14_BrN_5_O_5_, 508 g/mol, R*_f_* 0.88, yield 1124 mg, 74 %, Mp 220 ֯C. FTIR cm^-1^: 1697 (C=O), 3264 (N‒H isatin), 664 (C‒Br), 3219 (N‒H spiro), 1250 (C‒N), 1609 (C=C). ^1^HNMR (400 MHz, DMSO-*d*_6_): 6.25 (s, 1H, NH-pyra), 6.82 (s, 1H, H-4´), 6.98 (d, *J* = 8.5, 1H, H-7), 7.35-7.48 (m, 5H Ar-Ph), 7.42 (dd, *J* = 8.5, 2.1, 1H, H-6), 7.73 (1H, d, 𝐽 = 2.5Hz, H-3 DNPh), 7.86 (d, *J =* 2.1, 1H, H-4), 7.92 (1H, d, *J* = 8.5 Hz, H-6 DNPh), 8.48 (1H, dd, *J* = 8.5, 2.1 Hz, H-5 DNPh), 10.08 (s, 1H, NH). ^13^CNMR (75 MHz, DMSO-*d*_6_): 72.21 (C-3 spiro), 107.2 (C-4´), 115.65 (C-7), 122.13 (C-4), 125.18 (C-3a), 126.34 (C-4´´´), 127.05 (C-5), 127.86 (C-2´´´, 6´´´), 128.71 (C-3´´´, 5´´´), 129.04 (C-1´´´), 129.35 (C-2´´), 131.96 (C-6), 132.02 (C-7a), 132.27 (C-4´´), 135.28 (C-1´´), 136.57 (C-5´´), 137.87 (C-6´´), 139.74 (C-3´´), 151.45 (C-5´), 164.84 (C-2). Anal. calculated (%) For: C 51.99; H 2.78; N 13.78 Found: C 51.95; H 2.76; N 13.77.

**1.14 5-Bromo-2-oxo-5'-phenylspiro[indoline-3,3'-pyrazole]-1'(2'*H*)-carbothioamide (4n)** Brown solid, C_17_H_13_BrN_4_OS, 401 g/mol, R*_f_* 0.78, yield 1080 mg, 90 %, Mp 210 ֯C. FTIR cm^-1^: 1697 (C=O), 3264 (N‒H isatin), 668 (C‒Br), 3224 (N‒H spiro), 1250 (C‒N), 1608 (C=C), 1230 (C=S). ^1^HNMR (400 MHz, DMSO-*d*_6_): 6.22 (s, 1H, NH-pyra), 6.83 (s, 1H, H-4´), 6.94 (d, *J* = 8.32, 1H, H-7), 7.38 (dd, *J* = 8.31, 2.43, 1H, H-6), 7.52-7.64 (m, 3H Ar-Ph), 7.84 (d, *J* = 2.44, 1H, H-4), 10.15 (s, 1H, NH). ^13^CNMR (75 MHz, DMSO-*d*_6_): 69.21 (C-3 spiro), 106.2 (C-4´), 114.65 (C-7), 122.15 (C-4), 124.18 (C-3a), 124.34 (C-4´´´), 126.04 (C-1´´´), 128.05 (C-5), 129.71 (C-3´´´, 5´´´), 129.86 (C-2´´´, 6´´´), 131.96 (C-6), 133.02 (C-7a), 151.25 (C-5´), 165.79 (C-2), 171.36 (C-1´´). Anal. calculated (%) For: C 50.88; H 3.27; N 13.96; S 7.99. Found: C 50.86; H 3.25; N 13.93; S 7.95.

**1.15 5-Bromo***-****N*-methyl-2-oxo-5'-phenylspiro[indoline-3,3'-pyrazole]-1'(2'*H*)-carbothioamide (4o)** Yellow solid, C_18_H_15_BrN_4_OS, 415 g/mol, R*_f_* 0.83, yield 1003 mg, 79 %, Mp 218 ֯C. FTIR cm^-1^: 1697 (C=O), 3264 (N‒H isatin), 667 (C‒Br), 3222 (N‒H spiro), 1250 (C‒N), 1605 (C=C), 1228 (C=S). ^1^HNMR (400 MHz, DMSO-*d*_6_): 2.57 (s, 3H), 6.22 (s, 1H, NH-pyra), 6.83 (s, 1H, H-4´), 6.94 (d, *J* = 8.32, 1H, H-7), 7.38 (dd, *J* = 8.31, 2.43, 1H, H-6), 7.52-7.64 (m, 5H Ar-Ph), 7.84 (d, *J* = 2.44, 1H, H-4), 10.13 (s, 1H, NH). ^13^CNMR (75 MHz, DMSO-*d*_6_): 29.37 (C-3´´), 69.21 (C-3 spiro), 106.2 (C-4´), 114.65 (C-7), 122.13 (C-4), 124.18 (C-3a), 124.34 (C-4´´´), 126.08 (C-1´´´), 129.05 (C-5), 129.71 (C-3´´´, 5´´´), 129.86 (C-2´´´, 6´´´), 131.96 (C-6), 133.02 (C-7a), 151.25 (C-5´), 165.79 (C-2), 177.36 (C-1´´). Anal. calculated (%) For: C 52.06; H 3.64; N 13.49; S 7.72 Found: C 52.04; H 3.63; N 13.42; S 7.70.

**1.16 5-Bromo-1'-phenyl-5'-(thiophen-2-yl)-1',2'-dihydrospiro[indoline-3,3'-pyrazol]-2-one (4p)** Brick red solid, C_20_H_14_BrN_3_OS, 424 g/mol, R*_f_* 0.97, yield 574 mg, 73 %, Mp 206 ֯C. FTIR cm^-1^: 1697 (C=O), 3264 (N‒H isatin), 665 (C‒Br), 3224 (N‒H spiro), 1250 (C‒N), 1608 (C=C), 706 (C‒S). ^1^HNMR (400 MHz, DMSO-*d*_6_): 6.24 (s, 1H, NH-pyra), 6.91 (s, 1H, H-4´), 6.94 (d, *J* = 8.32, 1H, H-7), 7.38 (dd, *J* = 8.31, 2.43, 1H, H-6), 7.52-7.64 (m, 5H Ar-Ph), 7.71-7.89 (m, 3H Ar-Ph), 7.94 (d, *J* = 2.44, 1H, H-4), 10.12 (s, 1H, NH). ^13^CNMR (75 MHz, DMSO-*d*_6_): 70.21 (C-3 spiro), 104.2 (C-4´), 113.65 (C-7), 122.18 (C-3a), 122.34 (C-4), 125.74 (C-3´´, C-5´´), 127.05 (C-5), 127.28 (C-4´´), 127.86 (C-2´´´), 128.35 (C-2´´, C-6´´), 128.71 (C-3´´´), 131.96 (C-6), 132.02 (C-7a), 135.12 (C-1´´), 142.04 (C-1´´´), 147.62 (C-4´´´), 151.75 (C-5´), 162.84 (C-2). Anal. calculated (%) For: C 56.61; H 3.33; N 9.90; S 7.56 Found: C 56.59; H 3.31; N 9.87; S 7.54.

**1.17 5-Bromo-1'-(4-chlorophenyl)-5'-(thiophen-2-yl)-1',2'-dihydrospiro[indoline-3,3'-pyrazol]-2-one (4q)** Orange solid, C_20_H_13_BrClN_3_OS, 462 g/mol, R*_f_* 0.85, yield 1123 mg, 82 % Mp 210 ֯C, FTIR cm^-1^: 1697 (C=O), 3264 (N‒H isatin), 669 (C‒Br), 3223 (N‒H spiro), 1250 (C‒N), 1606 (C=C), 1171 (C‒S). ^1^HNMR (400 MHz, DMSO-*d*_6_): 6.24 (s, 1H, NH-pyra), 6.83 (s, 1H, H-4´), 6.94 (d, *J* = 8.32, 1H, H-7), 7.38 (dd, *J* = 8.31, 2.43, 1H, H-6), 7.48-7.67 (m, 4H Ar-Ph), 7.83 (d, *J* = 2.44, 1H, H-4), 7.64 (m, 3H Ar-Ph), 10.08 (s, 1H, NH). ^13^CNMR (75 MHz, DMSO-*d*_6_): 72.37 (C-3 spiro), 108.2 (C-4´), 113.65 (C-7), 123.18 (C-3a), 123.34 (C-4), 128.15 (C-5), 129.13 (C-4´´´), 129.32 (C-3´´´), 132.06 (C-6), 132.35 (C-2´´, C-6´´), 133.12 (C-7a), 134.28 (C-4´´), 135.2 (C-1´´), 136.86 (C-2´´´), 137.74 (C-3´´, C-5´´), 141.0 (C-1´´´), 151.26 (C-5´), 164.74 (C-2). Anal. calculated (%) For: C 52.36; H 2.86; N 9.16; S 6.99 Found: C 52.33; H 2.83; N 9.11; S 6.92.

**1.18 Bromo-1'-(2,4-dinitrophenyl)-5'-(thiophen-2-yl)-1',2'-dihydrospiro[indoline-3,3'-pyrazol]-2-one (4r)** Orange red solid, C_20_H_12_BrN_5_O_5_S, 514 g/mol, R*_f_* 0.97, yield 1247 mg, 81 %, Mp 220 ֯C. FTIR cm^-1^: 1697 (C=O), 3264 (N‒H isatin), 662 (C‒Br), 3220 (N‒H spiro), 1250 (C‒N), 1606 (C=C), 1171 (C‒S). ^1^HNMR (400 MHz, DMSO-*d*_6_): 6.83 (s, 1H, H-4´), 6.91 (s, 1H, NH pyra), 6.94 (d, *J* = 8.32, 1H, H-7), 7.38 (dd, *J* = 8.31, 2.43, 1H, H-6), 7.55 (1H, dd, 𝐽 = 8.8Hz, H-3 DNPh), 7.69 (1H, d, *J* = 8.8 Hz, H-6 DNPh), 7.71-7.89 (m, 3H Ar-Ph), 7.94 (d, *J* = 2.44, 1H, H-4), 8.30 (1H, dd, *J* = 8.8, 2.3 Hz, H-5 DNPh), 10.08 (s, 1H, NH). ^13^CNMR (75 MHz, DMSO-*d*_6_): 72.21 (C-3 spiro), 107.2 (C-2´), 115.65 (C-7), 125.18 (C-4), 126.34 (C-3a), 127.05 (C-5), 127.86 (C-4´´´), 128.71 (C-3´´´), 129.35 (C-2´´), 131.73(C-5´´´), 131.96 (C-6), 132.02 (C-7a), 132.27 (C-4´´), 135.28 (C-1´´), 136.57 (C-5´´), 137.87 (C-6´´), 139.74 (C-3´´), 142.04 (C-2´´´), 151.45 (C-1´), 164.84 (C-2). Anal. calculated (%) For: C 46.71; H 2.35; N 13.62; S 6.23 Found: 46.73; H 2.32; N 13.57; S 6.21.

**1.19 5-Bromo-2-oxo-5'-(thiophen-2-yl)spiro[indoline-3,3'-pyrazole]-1'(2'*H*)-carbothioamide (4s)** Brown yellow solid, C_15_H_11_BrN_4_OS_2,_ 407 g/mol, R*_f_* 0.87, yield 1085 mg, 89 %, Mp 208 ֯C, FTIR cm^-1^: 1697 (C=O), 3264 (N‒H isatin), 665 (C‒Br), 3224 (N‒H spiro), 1250 (C‒N), 1607 (C=C) , 1171 (C‒S). ^1^HNMR (400 MHz, DMSO-*d*_6_): 6.22 (s, 1H, NH-pyra), 6.83 (s, 1H, H-4´), 6.94 (d, *J* = 8.32, 1H, H-7), 7.38 (dd, *J* = 8.31, 2.43, 1H, H-6), 7.52-7.64 (m, 3H Ar-Ph), 7.84 (d, *J* = 2.44, 1H, H-4), 10.08 (s, 1H, NH). ^13^CNMR (75 MHz, DMSO-*d*_6_): 69.42 (C-3 spiro), 106.63 (C-4´), 114.87 (C-7), 122.53 (C-4), 124.24 (C-3a), 124.46 (C-4´´´), 126.25 (C-1´´´), 128.13 (C-5), 129.48 (C-3´´´, 5´´´), 129.97 (C-2´´´, 6´´´), 132.06 (C-6), 133.23 (C-7a), 151.28 (C-5´), 166.46 (C-2), 177.38 (C-1´´). Anal. calculated (%) For: C 44.23; H 2.72; N 13.76; S 15.74 Found: C 44.21; H 2.70; N 13.71; S 15.73.

**1.20 5-Bromo-*N*-methyl-2-oxo-5'-(thiophen-2-yl)spiro[indoline-3,3'-pyrazole]-1'(2'*H*) carbothioamide (4t)** Mustard solid, C_16_H_13_BrN_4_OS_2_, 421 g/mol, R*_f_* 0.80, yield 1058 mg, 84 %, Mp 210 ֯C. FTIR cm^-1^: 1697 (C=O), 3264 (N‒H isatin), 668 (C‒Br), 3218 (N‒H spiro), 1250 (C‒N), 1605 (C=C), 1171 (C‒S). ^1^HNMR (400 MHz, DMSO-*d*_6_): 2.57 (s, 3H), 6.22 (s, 1H, NH-pyra), 6.83 (s, 1H, H-4´), 6.94 (d, *J* = 8.32, 1H, H-7), 7.38 (dd, *J* = 8.31, 2.43, 1H, H-6), 7.52-7.64 (m, 5H Ar-Ph), 7.84 (d, *J* = 2.44, 1H, H-4), 10.08 (s, 1H, NH). ^13^CNMR (75 MHz, DMSO-*d*_6_): 27.46 (C-3´´), 69.42(C-3 spiro), 106.63 (C-4´), 114.87 (C-7), 122.53 (C-4), 124.24 (C-3a), 124.58 (C-4´´´), 126.25 (C-5´´´), 128.13 (C-5), 129.58 (C-3´´´), 129.97 (C-2´´´), 132.06 (C-6), 133.23 (C-7a), 151.28 (C-5´), 166.46 (C-2), 177.39 (C-1´´). Anal. calculated (%) For: C 45.61; H 3.11; N 13.30; S 15.22 Found: C 45.60; H 3.09; N 13.32; S 15.19.
